# Chloroform in Convulsions of Infants

**Published:** 1855-05

**Authors:** 


### CHLOROFORM IN CONVULSIONS OF INFANTS.

Dr. Brookhart, of Harrisonville, Missouri, in a letter to us reports cases of convulsions in infants successfully treated by the internal administration of chloroform. In one child the convulsions occurred every three hours. Dr. B. had it immersed in a warm bath when a spasm was about to come on and gave it six drops of chloroform. The child was permanently relieved by the treatment. Dr. B. has treated successfully three other cases in the same manner.

